# Thrombotic Microangiopathy Secondary to Capnocytophaga Sepsis: A Case Report

**DOI:** 10.7759/cureus.99194

**Published:** 2025-12-14

**Authors:** Mohamed Abdelsalam, Amin H Alayyan, Ahmed Elamin, Elizabeth S Goh, Adeel Bin Tariq

**Affiliations:** 1 Anesthesiology, Gateshead Health NHS Foundation Trust, Gateshead, GBR; 2 Internal Medicine, University Hospitals of Leicester NHS Trust, Leicester, GBR

**Keywords:** capnocytophaga, microangiopathic hemolytic anemia (maha), septic shock, splenic infarcts, thrombotic microangiopathy

## Abstract

*Capnocytophaga* species have been shown to cause a wide range of severe infections that can progress to severe sepsis. They also present a diagnostic challenge due to multiple factors, including non-specific symptoms and difficulty in microbiological detection due to slow growth. *Capnocytophaga* infection has been shown to be a potential trigger for thrombotic microangiopathy (TMA) through complement activation and endothelial injury that can trigger microthrombi formation. This presentation can sometimes mimic primary thrombotic thrombocytopenic purpura (TTP). Often, the combination of difficulty in establishing the underlying mechanism due to the unavailability of specialized diagnostic tests and rapid clinical deterioration following infection can lead to poor clinical outcomes. This is why early recognition and being able to differentiate between different causes of TMA is vital to enable clinicians to provide the correct treatment promptly. We present a case of TMA secondary to *Capnocytophaga* infection, treated solely with antimicrobial therapy.

## Introduction

*Capnocytophaga* is a capnophilic, facultatively anaerobic, gram-negative bacillus that inhabits the oral cavities of humans and domestic animals, primarily dogs [1]. The genus consists of nine species, six isolated from human oral flora, and these are associated with developing human oral bacterial infections. These include *C. gingivalis*, *C. ochracea*, *C. sputigena*, *C. granulosa*, *C. haemolytica*, and *C. leadbetteri*, which primarily infect immunocompromised patients [2].

The three remaining species that mainly inhabit the oral flora of canines are *C. canimorsus*, *C. canis*, and *C. cynodegmi*. These cause zoonotic infections through dog bite, lick, or even close exposure. Although more frequent and severe in immunocompromised patients, immunocompetent patients can still acquire a zoonotic infection [2,3].

Both zoonotic and non-zoonotic infections have frequently been reported in the literature and can become life-threatening by progressing into septic shock and multi-organ failure [2]. Possible sources of infections can include meningitis, cerebral abscess, infective endocarditis, pneumonia, cellulitis, septic arthritis, osteomyelitis, and endophthalmitis [2,4-6].

*Capnocytophaga canimorsus* is known to be one of the most common organisms to cause bacteremia and has been documented to cause all the above sites of infection. According to a nationwide study in the Netherlands, the annual incidence of *C. canimorsus* was 0.67 per million per year [2]. Documented risk factors for acquiring *Capnocytophaga* infections include advanced age, diabetes mellitus, chronic alcohol use, active hematological or solid organ malignancy, previous solid organ transplantation, liver disease, end-stage renal disease, and asplenia [7].

*Capnocytophaga* infection has also been associated with hematological complications such as disseminated intravascular coagulation (DIC) and thrombotic microangiopathy (TMA) [2,8], which accelerate multi-organ failure and significantly increase mortality [2,9]. In addition, *Capnocytophaga* species have been reported to be difficult to culture and acquire sensitivities due to slow growth [10]. This makes it difficult to deliver time-sensitive, lifesaving treatment. In this case, we present the case of a male patient in his 60s who presented with TMA secondary to *Capnocytophaga* infection with features resembling thrombotic thrombocytopenic purpura (TTP).

## Case presentation

A male in his 60s presented to the accident and emergency department with a six-day history of non-bloody diarrhea with around seven episodes per day, with one episode of vomiting. He also had generalized, intermittent, cramping abdominal pain of similar duration with severe intensity, intermittent low-grade fever, and a toothache. He denied any recent travel history, sick contacts, or unusual food consumption. Upon further history taking, it was noted that the patient’s pet dog had frequently come into close contact with the patient’s oral cavity.

His medical history includes recurrent dental abscesses, peripheral vascular disease, and polycythemia secondary to smoking. The patient is an ex-smoker with a 50 pack-year history, who is fully independent and lives with his wife and dog. His initial vitals revealed a blood pressure of 98/63 millimeters of mercury (mmHg) and a temperature of 35.7°C, with the rest of his vitals normal. His systematic review included bilateral foot pain. Apart from being fatigued, he was fully alert and conscious. On examination, he had mild tenderness to deep palpation in all abdominal quadrants.

His admission blood tests showed significant thrombocytopenia with acute kidney injury (AKI) and elevated inflammatory markers (Table 1). He was initially commenced on intravenous (IV) piperacillin-tazobactam for an infection source being either abdominal or dental.

**Table 1:** Blood test results on admission g: gram, mg: milligram, mL: milliliter, L: liter, µmol: micromole, mmol: millimole

Blood test (unit)	Result	Reference range
Hemoglobin (g/L)	163	130-180
Platelets (×10⁹/L)	34	150-450
Whtie cell count (×10⁹/L)	19.4	4-11
Neutrophils (×10⁹/L)	18.6	2-8
Lymphocytes (×10⁹/L)	0.5	1-4.8
C-reactive protein (mg/L)	349	0-5
Creatinine (µmol/L)	141	62-115
Estimated glomerular filtration rate (mL/min)	43	60-150
Urea (mmol/L)	13.9	2.5-7.8
Total bilirubin (µmol/L)	36	0-21

In less than 24 hours, he developed septic shock as his blood pressure dropped to 80/50 mmHg with poor response to fluid resuscitation, requiring intensive care unit (ICU) admission for vasopressor support and invasive monitoring. He became confused, developed a petechial rash over his nose, and became febrile. The blood gases showed a pH of 7.332 and a lactate of 4.7 millimoles per liter (mmol/L). Follow-up investigations (Table 2) revealed a decline in hemoglobin to 72 grams per liter (g/L), worsening platelets of 7x10^9^/L, deteriorating estimated glomerular filtration rate (eGFR) to 25 milliliters per minute (mL/min), and a procalcitonin of 25.7 microgram per liter (μg/L).

**Table 2:** Follow-up investigations g: gram, μg: microgram, mL: milliliter, L: liter

Blood test (unit)	Result	Reference range
Hemoglobin (g/L)	72	130-180
Platelets (×10⁹/L)	7	150-450
Estimated glomerular filtration rate (mL/min)	25	60-150
Procalcitonin (μg/L)	25.7	0.05-0.1

He continued on IV piperacillin-tazobactam with the addition of gentamicin. The bedside transthoracic echo was unremarkable, except for a hyperdynamic left ventricle with an ejection fraction of 60%.

The likely sources of sepsis were thought to be abdominal and/or dental, as there was gingival swelling. This prompted a computed tomography (CT) of the face, which revealed an upper right canine abscess, and an abdominal and pelvic (AP) CT was unremarkable. An acute surgical abdomen was excluded by surgical consultation.

Due to the decline in hemoglobin and renal function, worsening thrombocytopenia, and petechial rash, concerns of microangiopathic hemolytic anemia (MAHA) and particularly thrombotic thrombocytopenic purpura (TTP) were now entertained. Hematology consultation was sought in view of this, to which they suggested this combination of findings was likely thrombotic microangiopathy (TMA) related to sepsis rather than true TTP. He was transfused two units of platelets and packed red blood cells.

A hematology workup was done, and the results are shown in Table 3. Running further investigations, such as Willebrand factor-cleaving protease, also known as ADAMTS13, that would allow distinguishing thrombotic thrombocytopenic purpura from other thrombotic microangiopathies, was not feasible at that point.

**Table 3:** Hematology investigations g: gram, L: liter, µmol: micromole, mmol: millimole, IU: international unit, ng: nanogram, sec: second, INR: international normalized ratio

Blood test (unit)	Result	Reference range
Lactate dehydrogenase (IU/L)	1,320	125-243
Fibrinogen assay (g/L)	7.1	2.0-3.9
D-dimer (ng/L)	7,365	0-500
Prothrombin time (sec)	11.3	11.0-13.5
Activated partial thromboplastin time (sec)	27.2	23.9-33.8
INR ratio	0.9	0.8-1.2
Blood film (schistocytes)	Positive +++	N/A
Total bilirubin (μmol/L)	89	0-21
Conjugated bilirubin (μmol/L)	43	0-9

On day 3, blood cultures grew *Capnocytophaga* species; however, sensitivity and species identification were not done due to the difficulty of growing the organism. Fecal cultures taken on admission were unremarkable.

The provisional diagnosis was TMA due to sepsis secondary to *Capnocytophaga* species, with the source of infection being a dental abscess for which he underwent an incision and drainage of his right upper canine abscess.

The patient continued the same antimicrobial therapy, and improvements were reflected biochemically with a subsequent procalcitonin of 14.3 μg/L and being able to wean off vasopressors. His kidney function returned to baseline, and the confusion resolved. Platelet counts gradually improved on days 5 and 6. On the sixth day, the patient was successfully discharged to the ward and subsequently left the hospital.

## Discussion

*Capnocytophaga* infection presents a diagnostic challenge due to its non-specific clinical features and difficulty in microbiological detection. In this case, the organism was only identified after the onset of septic shock without identifying the exact species. Contact with the patient’s dog provided a plausible route for transmission, which has been reported in the literature [11]. Unfortunately, in this case, we could not identify the exact species.

Several methods can be used to identify *Capnocytophaga* species, including multilocus enzyme electrophoresis, serotyping of immunoglobulin A1 proteases, DNA probes, 16S rRNA PCR restriction fragment length polymorphism (RFLP) analysis, and 16S rRNA gene sequencing. Unfortunately, most of the methods mentioned can be time-consuming and costly. The 16s rRNA PCR RFLP, however, has been shown to generate accurate results in distinguishing *Capnocytophaga* species rapidly, and the method could easily be implemented in routine microbiology laboratories [1,12].

The presence of marked thrombocytopenia, biochemical evidence of hemolysis, and acute kidney injury raised concern for MAHA [13]. Confirmation of schistocytes on blood film and elevated lactate dehydrogenase (LDH) supported an intravascular hemolytic process. While MAHA indicates laboratory evidence of red blood cell fragmentation, TMA represents a broader clinical syndrome that includes MAHA, thrombocytopenia, and organ involvement due to microvascular thrombosis [13]. It has also been described as an umbrella term and can manifest through multiple etiologies such as malignancy, pregnancy, and medication [13].

The development of the above biochemical changes initially raised a strong suspicion of TTP. The presence of neurological changes, nasal petechial rash, and fever reflects the classic TTP pentad, which is rarely seen in full during clinical practice [14]. Although ADAMTS13 testing was not available and thus definitive exclusion of true TTP was not possible, the rapid improvement following initiation of antimicrobial therapy without plasma exchange supports an infection-associated secondary TMA mimicking TTP [11]. True TTP has a mortality above 90% without initiation of plasma exchange [14].

Hemolytic uremic syndrome (HUS) was considered less likely, as typical HUS mainly occurs in children less than five years old, and is commonly associated with primarily rapid renal deterioration secondary to Shiga toxin-associated *E. coli* (STEC), which was not supported by the patient’s microbiological results [15,16]. Atypical HUS (aHUS), which is primarily complement-mediated, usually presents with predominant renal involvement rather than extensive hematological abnormalities. In this patient, renal function responded well to sepsis management without the need for specific aHUS-directed therapy, making aHUS less likely [15]. Disseminated intravascular coagulopathy was deemed improbable due to normal clotting studies and paradoxically elevated fibrinogen levels [13,15].

Infection-related TMA is recognized as a secondary form triggered by systemic infection [13]. As bacteremia triggers complement activation meant to act as part of an innate immune response, it also results in unwanted endotheliopathy through endothelial cell dysfunction, resulting in increased cellular exocytosis of endothelial ultra-large von Willebrand factor (eULVWF) multimers [15,17]. The rise in multimers is thought to cause a relative insufficiency of ADAMTS13 [15]. Accumulation of multimers also stimulates excessive platelet aggregation, which triggers the coagulation cascade to form microthrombi in arterioles and capillaries of vital organs [15]. The subsequent clinical presentation may closely resemble TTP, which is a primary and severe ADAMTS13 deficiency, but would improve once the underlying infection is treated. Reports in the literature involving *Capnocytophaga* species describe similar TMA-like presentations previously named TTP-like syndrome resolving with antibiotic management alone, sometimes accompanied by splenic infarction [11,18]. This recurrent observation aligns with our findings and supports the hypothesis of an infection-induced transient microangiopathic process rather than true primary TTP. Table 4 highlights some key differences between TTP and infection-related TMA.

**Table 4:** Key differences between TTP and infection-related TMA TTP: thrombotic thrombocytopenic purpura, TMA: thrombotic microangiopathy

Diagnosis	TTP	Infection-related TMA
Trigger	Autoimmune/genetic	Sepsis/infection
ADAMTS13 activity	Severely low	Normal or mildly reduced
Renal involvement	Mild to moderate	Often more pronounced, but reversible
Response to antibiotics	None	Rapid improvement
Plasma exchange	Mandatory	Usually not required
Primary mechanism of microthrombi formation	ADAMTS13 deficiency	Endothelial injury and complement activation

This case emphasizes the need for maintaining a high index of suspicion for MAHA with rapidly falling platelet counts and evidence of hemolysis, as it can lead to early differentiation of TMA versus true TTP and thus initiating appropriate treatment early to have a favorable prognosis [11,15]. Additional research is warranted to improve understanding of the mechanisms involved in different causes of TMA and to support the development of appropriate diagnostic strategies.

## Conclusions

This case demonstrates that *Capnocytophaga* infection can cause severe sepsis even in immunocompetent individuals and may lead to complications such as TMA. A high degree of clinical suspicion, a thorough history, and an accurate differential diagnosis are essential to ensure appropriate management. In the presence of thrombocytopenia, hemolysis, and acute kidney injury, early consideration of TMA is crucial, as, depending on the underlying cause and available test results, the patient may require lifesaving plasma exchange, especially in cases of TTP. In addition, it is vital to educate both healthcare staff and the public about the potential health implications of zoonotic infections and the precautions needed when dealing with domestic and wild animals.
